# Coil Embolization of an Iatrogenic Arteriovenous Fistula between the Superior Mesenteric Artery and Vein: A Case Report

**Published:** 2019-01

**Authors:** Pezhman Farshidmehr, Mohammad Reza Zafarghandi, Alimohammad Sadat, Azadeh Sayarifard

**Affiliations:** 1 *Sina Hospital, Tehran University of Medical Sciences, Tehran, Iran.*; 2 *Center for Academic and Health Policy, Tehran University of Medical Sciences, Tehran, Iran.*

**Keywords:** *Arteriovenous fistula*, *Mesenteric artery, superior*, *Balloon occlusion*

## Abstract

Superior mesenteric arteriovenous fistulae are rare. A 32-year-old woman presented with abdominal pain. The angiography showed that the superior mesenteric vein was aneurysmal. The patient underwent coil embolization, during which a balloon catheter was inflated before the fistula for the protection of coil migration with a high blood flow. After the balloon inflation, one 8-mm and two 7-mm coils were deployed at the fistula site. The final angiography showed successful embolization with no visualization of the fistula and the aneurysmal vein.

## Introduction

Superior mesenteric arteriovenous fistulae (AVFs) are rare and commonly caused by trauma and iatrogenic injury.^1^ Most AVFs are asymptomatic. However, symptoms such as diarrhea, abdominal pain, ascites, pulmonary effusion, mesenteric ischemia, and bleeding have been reported.^1^^, ^^2^ If AVFs are left untreated, complications such as portal hypertension, congestive heart failure, and gastrointestinal tract hemorrhage may occur.^3^ Here, we present the case of a post-traumatic superior mesenteric AVF in a woman 10 years after the resection of a part of the small intestine. The patient consented to the publication of this case report.

## Case Report

A 32-year-old woman was referred to Sina Hospital, affiliated to Tehran University of Medical Sciences, with abdominal pain. The patient had a small bowel resection 10 years previously for bowel stenosis due to obstruction. 

A bruit was heard on the abdominal auscultation. The laboratory findings were normal. Computed tomography showed ectasia in the superior mesenteric vein secondary to an AVF.

**Figure 1:**
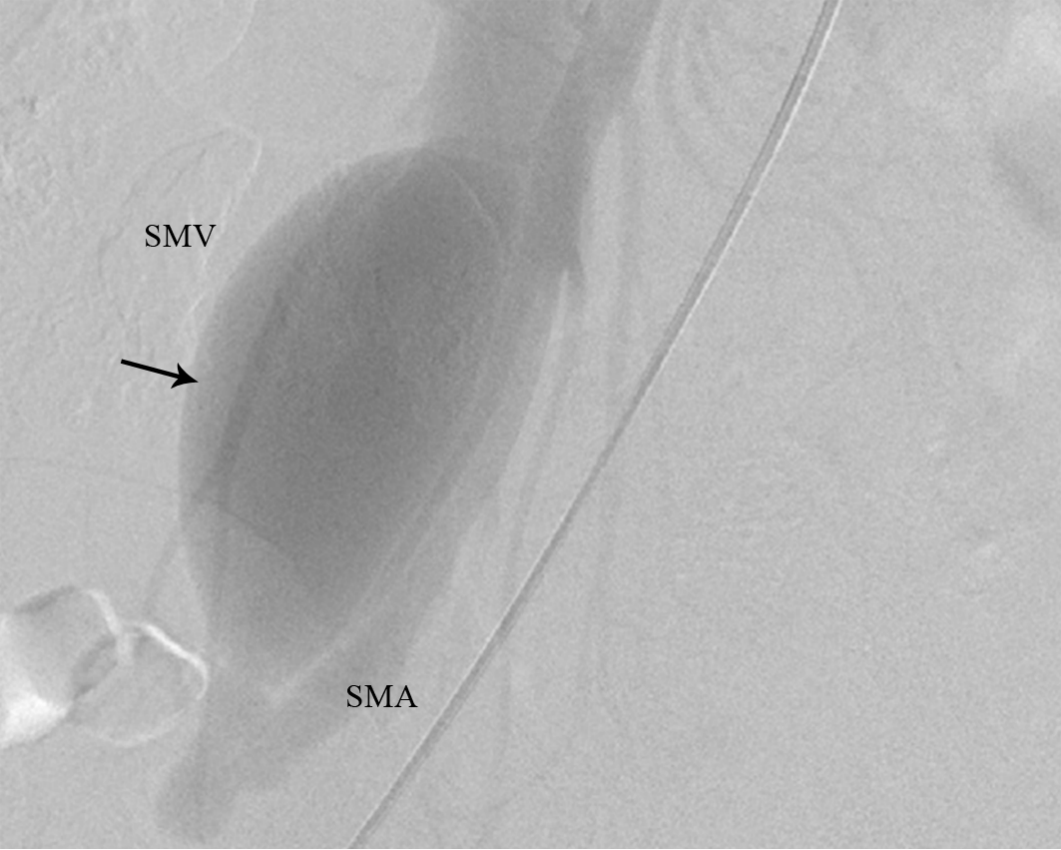
Lateral view of the abdominal angiography of the SMV and SMA before coil embolization (The arrow shows the site of the fistula before coil embolization.)

SMA, Superior mesenteric artery; SMV, Superior mesenteric vein

A 7-F guiding catheter (Cordis) was placed at the origin of the superior mesenteric artery via the right femoral artery. Then, the catheter was advanced into the superior mesenteric artery. Angiography was performed. There was a large fistula between the superior mesenteric artery and the superior mesenteric vein (Figure 1). The superior mesenteric vein was aneurysmal.

**Figure 2:**
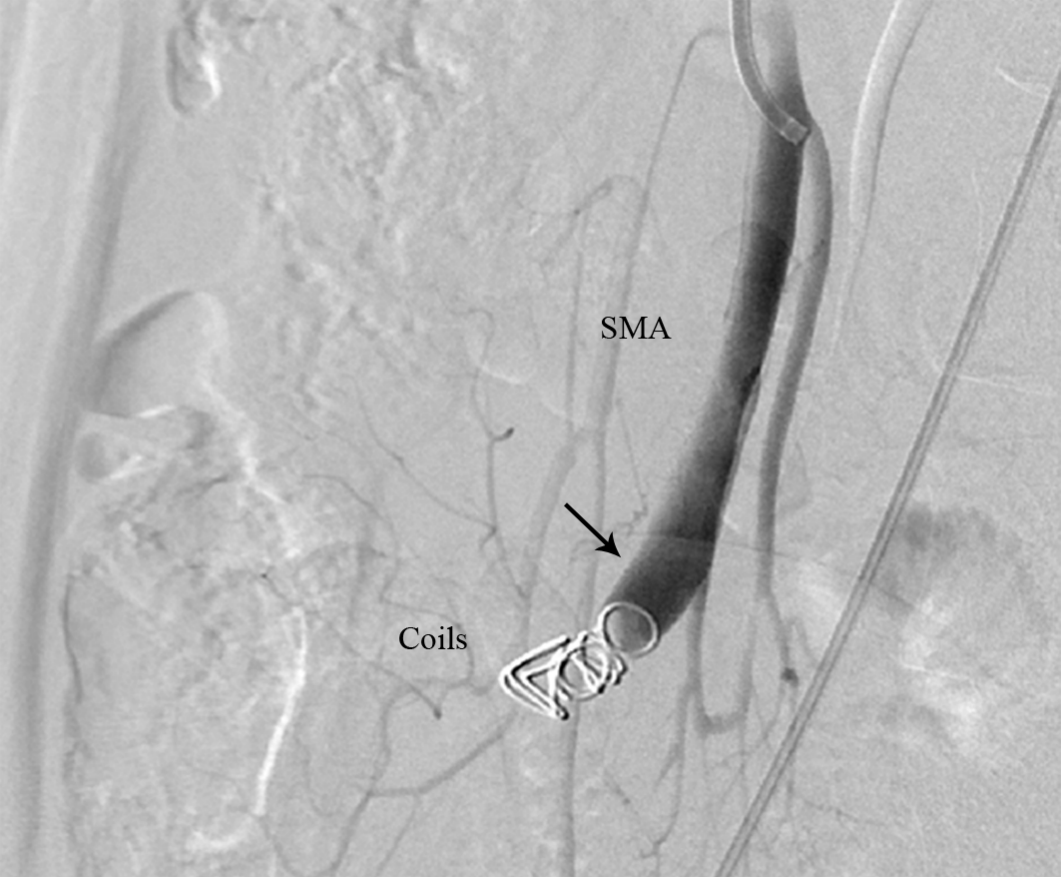
Lateral view of the abdominal angiography of the SMA after coil embolization (The arrow shows successful embolization with no visualization of the fistula and the aneurysmal vein.)

We decided to perform coil embolization (Cook) at the fistula site. Therefore, we inflated a 5-mm balloon catheter (Ev3, EverCross OTW balloon catheter) before the fistula to prevent coil migration with a high blood flow. After the balloon inflation, we deployed one 8-mm and two 7-mm coils at the fistula site. The final angiography showed successful embolization with no visualization of the fistula or the aneurysmal vein (Figure 2). 

SMA, Superior mesenteric artery

## Discussion

We presented the case of an AVF with abdominal pain. We used computed tomography to detect the abnormality and a selective angiography to determine the hemodynamics of the AVF.^4^ Delays in the treatment of AVFs may cause complications. Previous research has introduced a number of treatment methods, among which surgery is a traditional treatment approach in selected cases. 

Donell and Hudson^5^ reported the case of an AVF because of adhesions after a laparoscopic sterilization. The AVF was excised by surgery. The authors proposed an anatomic classification of such fistulae into H and U types for a better management. They also reviewed 11 cases of iatrogenic superior mesenteric AVFs which were treated surgically. 

Recently, endovascular treatment has been used more widely. Shintani et al.^4^ described a 37-year-old male patient with an AVF who presented with abdominal pain and diarrhea and had a history of Crohn’s disease and ileocecal resection. They performed endovascular treatment for the patient and reviewed 13 AVFs with endovascular treatment (coil embolization). Although this treatment has several potential problems, no mortality has been reported following this procedure.^5^

Wu et al.^1^ reported a post-traumatic superior mesenteric AVF in a 20-year-old man who presented with vomiting and severe epigastric pain and had a history of multiple stab wounds to the abdomen. Stent implantation was used as the treatment approach, and the complete closure of the fistula was confirmed on the follow-up angiography. 

Our patient had mild abdominal pain the day after the procedure. However, the pain subsided gradually and the patient was discharged in good general condition after 3 days. To prevent thrombosis in the superior mesenteric artery, we administered a heparin injection (1000 units/hour) and discharged the patient on oral warfarin for 3 months. At 9 months’ follow-up, he was healthy without any symptoms.

## Conclusion

We presented the case of a successful endovascular treatment with coil embolization of an AVF. Although endovascular treatment is minimally invasive and useful, further studies are required to identify the long-term complications of this method.

## Notes:

***This paper should be cited as: ***Farshidmehr P, Zafarghandi MR, Sadat A, Sayarifard A. Coil Embolization of an Iatrogenic Arteriovenous Fistula between the Superior Mesenteric Artery and Vein: A Case Report. J Teh Univ Heart Ctr 2019;14(1):37-39.
